# Application of *Trichoderma* Hz36 and Hk37 as Biocontrol Agents against Clubroot Caused by *Plasmodiophora brassicae*

**DOI:** 10.3390/jof8080777

**Published:** 2022-07-26

**Authors:** Yanli Zhao, Xingfu Chen, Jiasen Cheng, Jiatao Xie, Yang Lin, Daohong Jiang, Yanping Fu, Tao Chen

**Affiliations:** State Key Laboratory of Agricultural Microbiology, Hubei Key Laboratory of Plant Pathology, College of Plant Science and Technology, Huazhong Agricultural University, Wuhan 430070, China; yanlizhao733185@163.com (Y.Z.); cxf15055008862@163.com (X.C.); jiasencheng@mail.hzau.edu.cn (J.C.); jiataoxie@mail.hzau.edu.cn (J.X.); yanglin@mail.hzau.edu.cn (Y.L.); daohongjiang@mail.hzau.edu.cn (D.J.)

**Keywords:** clubroot, *Plasmodiophora brassicae*, rapeseed, *Trichoderma guizhouense*, *Trichoderma koningiopsis*, biological control, germination of resting spores, clubroot development

## Abstract

Clubroot, a soil-infective disease caused by *Plasmodiophora brassicae*, is a serious disease affecting cruciferous plants around the world. There is no effective control measure to completely remove this pathogen from fields after infection. Here, we screened and identified two strains (Hz36, *Trichoderma guizhouense*; Hk37, *Trichoderma koningiopsis*) of *Trichoderma* from the gall of clubroot in rapeseed fields with biocontrol potential for clubroot. The fermentation broth of Hz36 could significantly inhibit the germination of resting spores of *P. brassicae*, and promote the seed germination and root growth of rapeseed. The biocontrol efficiency of Hz36 strain on clubroot for rapeseed and *Arabidopsis* *thaliana* was 44.29% and 52.18%, respectively. The qPCR results revealed that strain Hz36 treatment could significantly reduce the content of *P. brassicae* in root cells, and paraffin section analysis revealed that it could delay the development of *P. brassicae*. Strain Hk37 showed similar effects to strain Hz36, whose biocontrol efficiency of clubroot could reach 57.30% in rapeseed and 68.01% in *A*. *thaliana*. These results indicate that strains Hz36 and Hk37 have the potential for the biocontrol of clubroot.

## 1. Introduction

Clubroot, a disease caused by the soil-living and obligate biotrophic protist *Plasmodiophora brassicae*, is the most devastating disease of cruciferous crops worldwide [1,2]. Clubroot development is characterized by the formation of club-shaped galls on the roots of the infected plants, which interrupts the absorption of water and nutrients by plants, resulting in wilting, stunting, yellowing and premature senescence symptoms and finally average of 10–15% reduction in yield on a global scale, with 60–90% yield loss in the severely infested fields when planted susceptible canola/rapeseed cultivars [1].

The control of clubroot has always been an issue of great concern worldwide. The resting spores of *P. brassicae* are easily transmitted through contaminated soil, including farm machinery, boots, grazing animal hooves, infected transplants, and surface floodwater [3]. These spores are robust and can persist in the soil for more than 15 years [1]. Commonly used strategies for the efficient control of this disease include crop rotation, increasing soil pH, improvement of drainage conditions, application of fungicide, adoption of resistant host plants and biological control [4]. Genetic resistance is the most effective and economical approach for clubroot management, and several resistant cultivars against clubroot have been reported [5,6,7]. However, new pathotypes of the pathogen will keep emerging [8], and not all R genes are active against the new pathotypes. In addition under high clubroot pressure, these measures are generally not effective [9].

Due to the lack of effective control measures against *P. brassicae*, it is urgent to explore novel control strategies. Biological control measures are particularly effective to reduce soil-borne pathogens. In recent years, research on biological control against clubroot has attracted increasing attention. *Bacillus subtilis* XF-1 is a well-characterized biological control agent in China, which has the most obvious positive effect at the early stage of seedling development. In the field, *B. subtilis* XF-1 can reduce the disease index by about 17% [10]. *Bacillus amyloliquefaciens* strain QST713 has been registered for commercial use in many countries [11], and applied to control *P. brassicae* in Canada [12]. Recently, two new strains *Bacillus velezensi* F85 and *B. amyloliquefaciens* T113 described as a biocontrol agents against *P. brassicae* [13]. The fungal genus *Trichoderma* comprises several species that have been well studied as biological control agents against *P. brassicae* [14,15]. In greenhouse pot experiments, the biocontrol efficiency of *T. harzianum* strain T4 against *P. brassicae* in Chinese cabbage could reach up to 79% [16]. Another study has also demonstrated the biocontrol effect of *T. harzianum* strain LTR-2 against *P. brassicae* in Chinese cabbage in the field, which was found to reduce the disease incidence from 96.7% to 51.3% [17]. The application of biological control measures could help to reduce soil-borne pathogens in particular. To improve control strategies of clubroot, we still need to explore new biocontrol agents.

The objectives of this study were to: (1) isolate effective antagonistic fungus from the symptomatic and asymptomatic roots of rapeseed infected with *P. brassicae* in severely infected fields; (2) screen, identify and characterize the candidate strains; (3) evaluate the efficacy of strains under greenhouse conditions in Arabidopsis and rapeseed; and (4) examination candidate strains affect the *P. brassicae* development and host growth. The goal was to identify new promising candidates to be used as biocontrol of clubroot.

## 2. Materials and Methods

### 2.1. Isolation of Fungal Strains inside the Rapeseed Root

The symptomatic and asymptomatic roots of rapeseed infected with *P. brassicae* in severely fields in Zhijiang City, Hubei Province. Root samples were soaked in 75% ethanol for 3 min, and washed with sterile water 6 times. The root epidermis was removed with a sterilized knife and the root was cut into small pieces. Small pieces of root were inoculated on potato-dextrose agar (PDA; 200 g potato boiled and filtered, 20 g dextrose, 10 g agar, 1000 mL distilled water) in a 9 cm Petri dish, grown at 25 °C in darkness. In order to suppress bacterial growth, 50 mg/L of cephalosporin was added to the PDA agar. Hyphal-tip from the growing edge of colonies cultured for 2–3 days at 25 °C was transferred to PDA to obtain pure cultures. The isolated fungal species were maintained in glycerol stock at −80 °C.

### 2.2. Morphological Characterization of Strains Hz36 and Hk37

Strains Hz36 and Hk37 were grown on PDA plates for two weeks in a growth chamber at 25 °C with a 12 h light/dark cycle. To assess and describe the structure and morphology of conidiophores, mycelia were taken from the edge of conidiogenous pustules or fascicles. It took 14 days for conidial induction to be observed. Microscopic morphologies such as conidia and conidiophore were observed using an optical microscope (Nikon Eclipse 90i or Olympus BX63, Tokyo, Japan).

### 2.3. Fungus DNA Extraction, PCR and ITS Sequencing

Strains were grown on PDA in 9 cm Petri dishes for 3 days at 25 °C. DNA was extracted from fresh mycelia using the CTAB method as described by Turner et al. [18]. The ITS1/ITS4 primers are used for amplification of the ITS region of the endophytic fungi (Tables S1 and S4). The PCR product was sequenced and taxonomy of each fungus was determined by Ribosomal Database Project at Michigan State (http://rdp.cme.msu.edu/classifier/classifierServlet (accessed on 14 July 2022)). For the phylogenetic analysis, DNA sequences of a-actin (ACT), calmodulin (CAL), internal transcribed spacers rDNA regions (ITS), and translation elongation factor 1-α (TEF1) were used in the phylogenetic analyses. The primers (Table S4) used included Tact1 and Tact2 for ACT [19,20], CAL-228F and CAL-737R for CAL [19], ITS5 and ITS4 for ITS [21], and EF1-728F and EF1-1R for TEF1 [19,22]. PCR reactions were performed using 2 ×Mix and protocols described previously [23,24,25]. PCR products were cleaned and sequencing was performed at the DNA sequencing facility.

### 2.4. Phylogenetic Analyses of Hz36 and Hk37 Strains

Sequences were aligned with MAFFT [26] as the external sequence alignment tool and RAxML [27] as the tree estimator. The maximum likelihood (ML) analysis was performed with all sequences, first with each gene/locus separately and then with the concatenated datasets. Phylogenetic analysis with (*ACT*-*TEF1*-*CAL*-ITS) and (*TEF1*-*CAL*-*ACT*) was carried out to illustrate the position of Hz36 and Hk37, respectively.

### 2.5. Fungal Spore Preparation

The conidia on the surface of the colony were flooded with sterile ddH_2_O. The solution with conidia was then filtered through sterile lens cleaning tissue paper to remove any hyphal fragments present. The number of spores can be counted using a hemocytometer, diluted to 1 × 10^7^ spores/mL in ddH_2_O for use.

### 2.6. Plant Materials, P. brassicae Inoculation and Growth Conditions

The *Arabidopsis thaliana* ecotype Columbia (Col-0) and *Brassica napus* HuasFhuang 4 were used. Plants were grown in soil in a growth chamber at 22 °C and 75% humidity with a 16-h-light/8-h-dark photoperiod.

The resting spores of *P. brassicae* were extracted from clubroot galls [28], surface disinfested by freshly prepared 2% chloramine-T solution at room temperature for 20 min, washed twice with sterile water, adjusted to a concentration of 1.0 × 10^7^ spores per mL, and then stored at 4 °C for later use.

Two-week-old plants were used for pathogen infection. Plants were inoculated with 1 mL of the resting spore suspension (1.0 × 10^7^ spores per mL) through the soil around each plant. The phenotype of Col-0 and Huashuang 4 was verified at 21 days and 30 days post inoculation (dpi), respectively. The disease severity in Arabidopsis was assessed using a scoring system of 0–4 modified from the report of Siemens et al. [29]. A score of 0 indicated no disease; 1, very small galls mainly on lateral roots that did not impair the main root; 2, small galls covering the main root and a few lateral roots; 3, medium to large galls, also on the main root, and 4, severe galls on lateral root, main root or rosette, with fine roots completely destroyed. Disease index (DI) was calculated using the five-grade scale according to the formula: DI = (1n_1_ + 2n_2_ + 3n_3_ + 4n_4_) × 100/4N_t_, where n_1_–n_4_ is the number of plants in the indicated class and N_t_ is the total number of plants tested. For each biological experiment, at least 15–30 plants were analyzed. Similar results were obtained from three independent biological experiments. The disease severity in *rapeseed* was assessed using a scoring system of 0–9 modified from the report of Zhang et al. [30]. A score of 0 indicated no disease, healthy roots; 1, single gall on lateral roots; 2, several small galls on lateral roots, plant still healthy; 3, mild galling on taproot, several small galls on lateral roots; 4, moderate galling on taproot, several small galls on lateral roots; 5, moderate galling on taproot, several large galls on lateral roots; 6, severe galling on taproot, many large galls on laterals; 7, severely galled, several healthy roots remaining; 8, severely galled, few healthy roots present; 9, severely galled, no healthy roots present. The following formulas were used for calculating biocontrol efficacy (CE): CE = (DI_control_ − DI_treatment_)/DI_control_ × 100%, where DI_control_ is the disease index of control, DI_treatment_ is the disease index of fungus treatment.

### 2.7. Germination Assay of P. brassicae Resting Spores

The effect of fermentation broth on the germination of *P. brassicae* resting spores was assessed in the presence of host root exudates (*B. napus* seedlings) according to the previously described method [31,32]. Hz36 and Hk37 were cultured in PD liquid medium (PDA medium without agar) for 7 days (25 °C, 180 r min^−1^). Then, the fermentation broth of Hz36 and Hk37 was passed through a 0.22-μm filter membrane under sterile conditions to collect the fermentation products. The seeds of cultivar Huashuang 4 were surface-sterilized with household sodium hypochlorite and pregerminated on a wet-filter paper in Petri dishes. After one week, the root exudates were collected through a 0.22-μm filter membrane and stored at 4 °C. The resting spores of *P. brassicae* (final concentration 1.0 × 10^7^/mL) incubated with equal volumes of mixed fermentation broth and root exudates (each sample added 500 μL fermentation broth and 500 μL root exudates), covered with tin foil and kept it in the growth chamber for 3 and 6 days. PD liquid medium was used instead of fermentation broth as the negative control. Centrifugation of the sample at 8000 rpm for 1~2 min and discarded the supernatant, resuspended in 300 μL sterile water. A 100 µL aliquot of the spore suspension was mixed and stained with an equal volume of 2 µg/mL 4′-6-diamidino-2-phenylindole-dihydrochloride (DAPI, Sigma-Aldrich, Shanghai, China) in 0.1% (*v*/*v*) dimethylformamide, and 5 µL of the mixture was transferred onto a glass slide and covered with an 18 mm × 18 mm cover glass. The total number of spores and the number of spores with a nucleus in one field of view were counted under UV excitation, using a fluorescent microscope at 400× magnification. Approximately 200 spores were examined from each sample and each sample was repeated three times [33].

### 2.8. Plant RNA Isolation, Plant DNA Isolation and qPCR Analysis

For quantification the expression level of *P. brassicae* in infected roots, the *A. thaliana* and *B. napus* root galls were washed with sterilized water, and then finely ground in liquid nitrogen. Total RNA was isolated from the roots using TRIzol reagent according to manufacturer’s instructions and quantified with a nano drop spectrophotometer. First-strand cDNA was prepared using oligo (dT)18 primer, and the resulting product was directly used as template for qPCR. For quantification the *P. brassicae* DNA content in infected roots, total DNA of *A. thaliana* and *B. napus* root galls were extracted using the cetyl trimethyl ammonium bromide (CTAB) method [34], and 2.5 ng total DNA was directly used as template for qPCR [35]. qPCR was performed using i-Taq Universal SYBR Green Super mix (Bio-Rad, Hercules, CA, USA) and a CFX96 real time PCR system (Bio Rad). The following cycling conditions were used: 95 °C for 30 s, 95 °C for 5 s, 60 °C for 15 s, and 72 °C for 12 s. The reaction was performed for 40 cycles, followed by a step at 72 °C for 5 s. Each amplification used three technical replicates, and the results were averaged to give the value for a single biological replicate. GenBank accession numbers of *P. brassicae* target actin gene is AY452179.1, *A. thaliana* actin gene is AT3G18780, respectively. *A. thaliana* actin gene was used as an internal control for data normalization [34]. The primer sequences are provided in Table S4.

### 2.9. Microscopic Analysis

Microscopy was performed using the following protocol. For toluidine blue dye microscopy, the preparation of samples was performed by Wuhan Bo Er Fu Biological Company. The roots were cross sectioned, treated with FAA fixative, washed and dehydrated, and finally paraffin-embedded into sections, stained with toluidine blue. The samples were subsequently observed using Nikon light microscopy (Nikon Eclipse 90i or Olympus BX63, Tokyo, Japan). For electron microscopy (TEM) analysis, roots were fixed in 2.5% glutaraldehyde for 4 h, and subsequently postfixed in 1% osmium tetroxide for 3 h, washed, dehydrated through an ethanol series, and embedded in London resin white. Ultrathin sections were examined through TEM (HITACHI, H-7000, Tokyo, Japan).

### 2.10. Seed Treatment

About 2000 seeds of cultivar Huashuang 4 were sterilized with 2% sodium hypochlorite, followed by washing with sterile water to remove surface contaminants. Then the seeds were soaked in the 100 mL fermentation filtrate (passed through 0.22-μm membrane filters) of strains Hz36 or Hk37 for 6 h. A piece of sterilized filter paper was spread on the bottom of a 9 cm Petri dish, and 100 treated seeds were placed on it evenly. Sterile water was used as the blank control. Three replicates were set for each group, and all the seeds were placed in the growth chamber (culture conditions set: 23 °C, 12 h light, 12 h dark, 70% relative humidity) to grow. About 5 mL of sterile water was added to the Petri dish every day, and the germination rate of seeds was counted for consecutive 7 days. At the same time, the root length of rapeseed on the 7th day and 12th day was analyzed.

### 2.11. Statistical Analysis

Two-tailed Student’s *t*-test and one-way ANOVA followed by Tukey’s multiple comparisons were conducted using Prism 8. All experiments were repeated for three times.

## 3. Results

### 3.1. Identification of Strains Hz36 and Hk37

In order to screen novel biocontrol agents applicable in clubroot disease management, 44 endophytic fungal strains were isolated from the symptomatic and asymptomatic roots of rapeseed infected with *P. brassicae* in severely infected fields; 35 out of 44 belong to *Trichoderma* (Table S1), divided into seven species. *Trichoderma* spp. are commercially employed in many control agents against a diverse group of plant pathogens [14,15]. *Trichoderma* can effectively reduce the incidence of clubroot disease in Chinese cabbage by regulating the rhizosphere microbial community [16,17]. We selected 8 different candidate *Trichoderma* strains (covering six species) to test their biocontrol properties against clubroot in Arabidopsis in pot experiments (Table S1). The results suggested that two candidate strains showed over 50% biocontrol efficacy, which were designated as Hz36 and Hk37.

**Figure 1:**
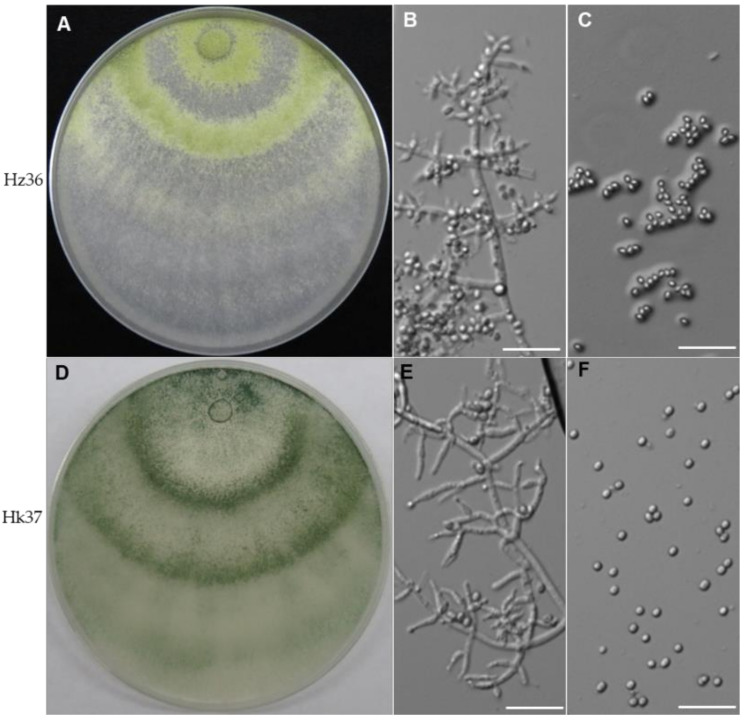
Identification of Hz36 and Hk37 strains. (**A**–**C**) Colony morphology (**A**), conidiophore (**B**), and conidia (**C**) of Hz36 strain. (**D**–**F**) Colony morphology (**D**), conidiophore (**E**), and conidia (**F**) of Hk37. The morphology was observed for the strain grown on PDA medium for 7 days at 25 °C with 12 h light/dark cycle, scale bar = 20 μm.

Strain Hz36 was grown on PDA for 7 days at 25 °C with 12 h light/dark cycle. The culture showed cottony and yellow-green conidia with the formation of thick and dense concentric rings (Figure 1A). Conidiophores were hyaline, smooth-walled and verticillate, forming a more or less pyramidal structure (Figure 1B), and conidia were one-celled, globose with a diameter of 2–3 μm, smooth-walled, and pale yellow-green (Figure 1C). According to the morphological characteristics of the observed colonies, it could be identified as *Trichoderma*. The ITS sequence of Hz36 showed a 99.38% identity with that of *Trichoderma guizhouense,* which belongs to the *Harzianum* clade. In order to identify strain Hz36 further, the *ACT*, *TEF1*, *CAL* and ITS sequences of 40 taxa of the *Harzianum* clade were downloaded from the GenBank and aligned with those of Hz36 for maximum likelihood (ML) phylogenetic analysis (Table S2). The gene regions were combined. Sequences from the four gene regions were combined for a total of 2477 aligned characters (752, 651, 454 and 620 characters for *the ACT*, *TEF1*, *CAL* and ITS, respectively). *T. aggressivum* CBS 100525 and *T. aggressivum* CBS 433.95 were selected as outgroups. Hz36 was the closest to *T. guizhouense* (Figure S1). Taken together, strain Hz36 was identified as *T. guizhouense.*

The Hk37 strain was grown on PDA for 7 days at 25 °C with a 12 h light/dark cycle. Dark green conidia were formed in concentric rings (Figure 1D). Conidiophores showed a distinct main axis with a width of about 3 μm, and the included angle was slightly less than 90° (Figure 1E). Conidia were globose, smooth-walled and pale green (Figure 1F). Based on the morphology, Hk37 could be identified as *Trichoderma*. The ITS sequence of Hk37 showed a 99.68% identity with *Trichoderma koningiopsis.* In order to identify strain Hk37 further, the *TEF1*, *CAL* and *ACT* sequences of 25 taxa of the *T. viride* clade were downloaded from the GenBank and aligned with those of Hk37 for ML phylogenetic analysis (Table S3). The gene regions were combined. Sequences from the three gene regions were combined to for a total of 1697 aligned characters (614, 372 and 711 characters for *TEF1*, *CAL* and *ACT*, respectively). *T. hamatum* DAOM 167057 was selected as the outgroup. Hk37 was the closest to *T. koningiopsis* (Figure S2). Therefore, Hk37 could be identified as *T. koningiopsis.*

### 3.2. Biocontrol Effects of Hz36 and Hk37 on A. thaliana Clubroot Disease

**Figure 2:**
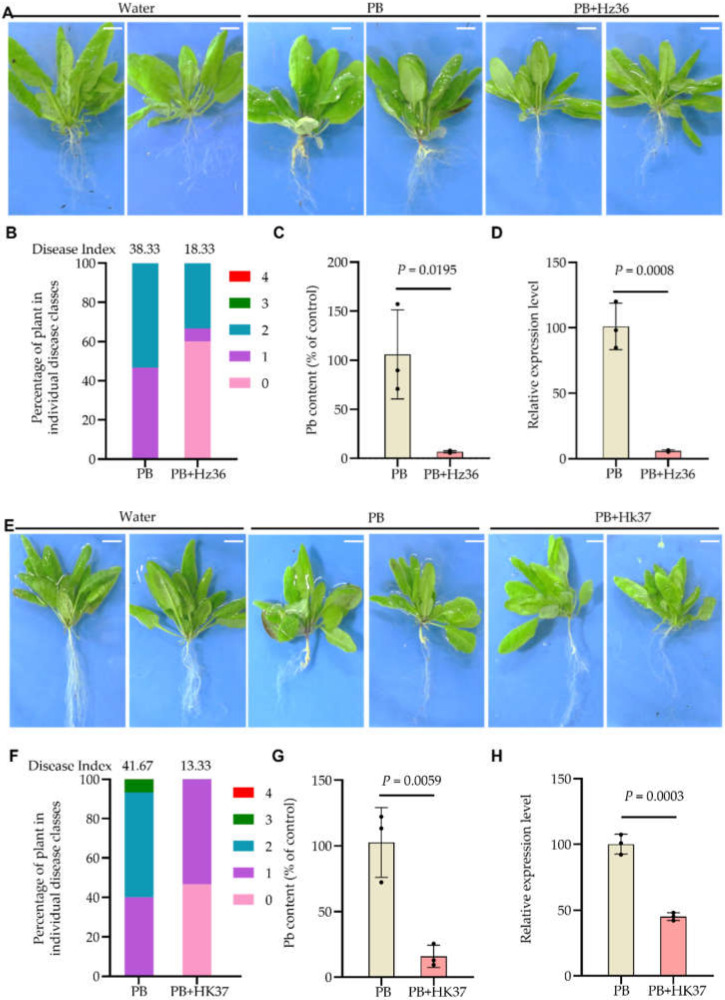
Phenotypes and *P. brassicae* DNA content in the roots of *A. thaliana* treated with strains Hz36 and Hk37. (**A**) Phenotype of *A. thaliana* infected by *P. brassicae* alone or together with Hz36for 21 days. (**B**) Disease index of samples in A. (**C**) qPCR analysis was performed to assess *P. brassicae* DNA content between inoculated with *P. brassicae* alone or co-inoculated with *P. brassicae* and Hz36, the sample of A, 3 plant roots were taken as a mixed sample. (**D**) qPCR analysis was performed to assess the expression levels of *P. brassicae* at 21 days after inoculation in the sample of A. Three plant roots were taken as a mixed sample. (**E**) Phenotype of *A. thaliana* treated with or without Hk37 after inoculated with *P. brassicae* for 21 days. (**F**) Disease index of samples in D. (**G**) *P. brassicae* DNA content in the samples of E detected by qPCR, 3 plant roots were taken as a mixed sample. (**H**) qPCR analysis was performed to assess the expression levels of *P. brassicae* at 21 days after inoculation in the sample of E, 3 plant roots were taken as a mixed sample. Water indicates the plants inoculated with as a negative control; PB represents the plants inoculated with *P. brassicae* as a positive control; PB+Hz36 indicates the plants co-inoculated with *P. brassicae* and strain Hz36; PB+Hk37 represents the plants co-inoculated with *P. brassicae* and strain Hk37, scale bar = 1 cm. The student’s two-tailed *t*-test was performed for comparison of means between two data points in C, D, G and H, and the data are shown as mean ± s.d. (*n* = 3 biological replicates), black dots show three biological replicates. Exact *p*-values for all comparisons are shown in the source data. Experiments were repeated three times with similar results.

In order to explore the effect of Hz36 and Hk37 on the growth, development and pathogenic process of clubroot, we first examined the phenotypes and determined the cDNA content of *P. brassicae* in the roots of *A. thaliana* Col-0 after strain Hz36 and Hk37 treatment (Figure 2). Typical galls were formed on the roots of Col-0 infected with *P. brassicae* (PB), resulting in few rootlets (Figure 2A), and the disease index was 38.33 (Figure 2B). Interestingly, more than half of Col-0 co-inoculated with *P. brassicae* and Hz36 (PB+Hz36) did not form any gall, and the root still developed with plentiful lateral roots (Figure 2A). The disease index was 18.33 (Figure 2B), and the overall biocontrol efficiency was 52.18%. To evaluate *P. brassicae* production in the galls, the relative amount of *P. brassicae* DNA in total root-extracted DNA was evaluated through qPCR (Figure 2C). The results indicated that Hz36 treatment significantly reduced pathogen density in the roots (Figure 2C). To evaluate *P. brassicae* production in the galls, the actin gene expression levels of *P. brassicae* were measured using qPCR (Figure 2D). The results suggested that *P. brassicae* production was significantly reduced after Hz36 treatment. We also examined the phenotypes and determined the *P. brassicae* DNA content in the roots of Col-0 after Hk37 treatment. Similar to Hz36 treatment, Col-0 control formed typical galls, resulting in few rootlets (Figure 2E), and the disease index was 41.67 (Figure 2F). However, in Col-0 co-inoculated by *P. brassicae* and Hk37 (PB+Hk37), no obvious gall was formed; the root system still developed with plentiful lateral roots (Figure 2E); and the disease index was 13.33 (Figure 2F). The biocontrol efficiency was 68.01%. qPCR results showed that after PB+Hk37 treatment, the *P. brassicae* content and production was very low (Figure 2G,H). Taken together, Hz36 and Hk37 increased resistance to clubroot in the model plant *A. thaliana*, suggesting that these two strains have great potential for the biocontrol of clubroot disease.

### 3.3. Biocontrol Effects of Hz36 and Hk37 on Rapeseed Clubroot Disease

**Figure 3:**
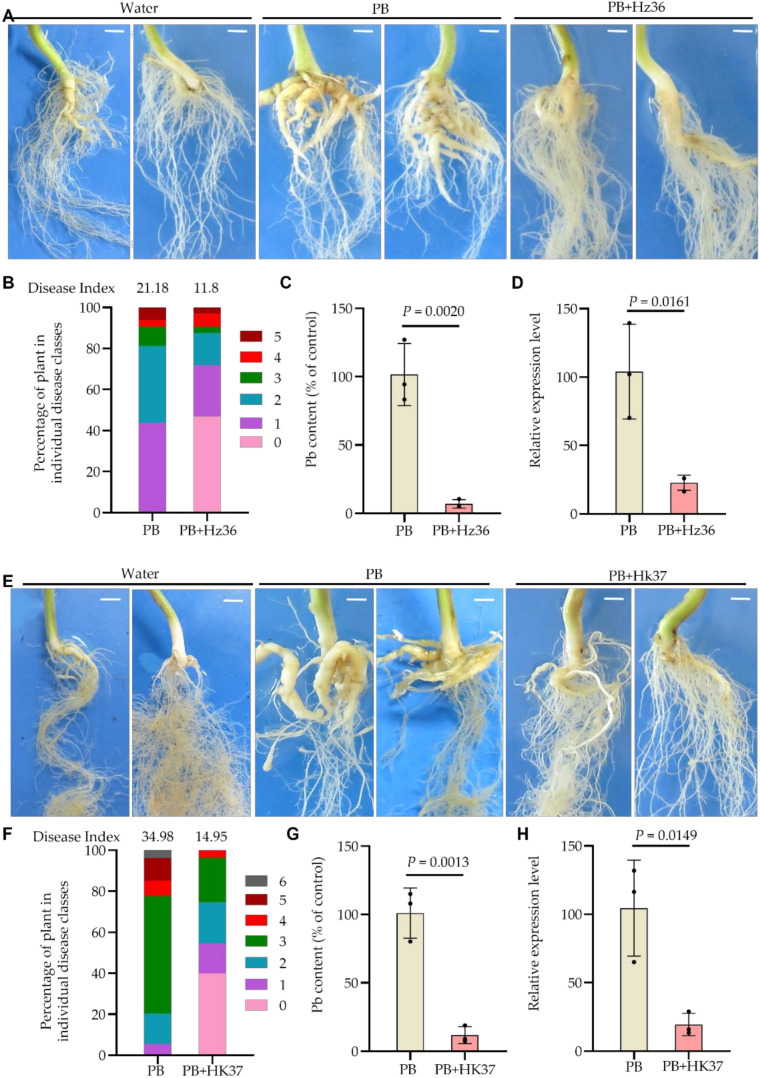
Phenotypes and *P. brassicae* DNA content in the roots of rapeseed treated with Hz36 and Hk37 strains. (**A**) Phenotype of rapeseed infected by *P. brassicae* alone or together with Hz36 for 30 days. (**B**) Disease index of samples in A. (**C**) *P. brassicae* DNA content in the samples of A detected by qPCR. (**D**) qPCR analysis was performed to assess the expression levels of *P. brassicae* at 21 days after inoculation in the sample of A. Three plant roots were taken as a mixed sample. (**E**) Phenotype of rapeseed infected by *P. brassicae* alone or together with Hk37 for 30 days. (**F**) Disease index of samples in E. (**G**) *P. brassicae* DNA content in the samples of E detected by qPCR. Water indicates the plants inoculated with water as a negative control; PB represents the plants inoculated with *P. brassicae* as a positive control; PB+Hz36 indicates the plants co-inoculated with *P. brassicae* and strain Hz36; PB+HK37 represents the plants co-inoculated with *P. brassicae* and strain HK37, scale bar = 1 cm. The student’s two-tailed t-test was performed for comparison of means between two data points in (**C**,**D**,**G**,**H**), and the data are shown as mean ± s.d. (*n* = 3 biological replicates, 3 plant roots were taken as a mixed sample), black dots show three biological replicates. Exact *p*-values for all comparisons are shown in the source data. Experiments were repeated three times with similar results.

In order to confirm the biocontrol effect of Hz36 and Hk37 on clubroot in cruciferous crops, we co-inoculated the rapeseed cultivar Huashuang 4 with P. brassicae (PB) and Hz36 (PB+Hz36) or Hk37 (PB+Hk37). The rapeseed roots infected by PB alone (control) showed the formation of typical galls (Figure 3A), with a disease index of 21.18 (Figure 3B). However, after treatment with PB+Hz36, 46.875% of the roots did not form galls with a disease index of 11.8 (Figure 3A,B), and the pathogen density in the roots was significantly reduced compared with that in the control (Figure 3C,D). The biocontrol efficiency was 44.29%. These results suggested that Hz36 has strong resistance to P. brassicae. The same experiment was also performed on strain Hk37 and similar results were obtained. Typical galls were also formed on rapeseed roots of the control (Figure 3E), and the disease index was 34.98 (Figure 3F). Over 40% of rapeseed roots treated by PB+Hk37 did not form any gall (Figure 3E), with a disease index of 14.95 (Figure 3F). The overall biocontrol efficiency was 57.3%. The qPCR results revealed that the P. brassicae content in the galls was significantly reduced compared with tplant roots were taken as a mixed samplehat in the control (Figure 3G,H). These results demonstrated that Hk37 also has strong resistance to P. brassicae. Taken together, Hz36 and Hk37 may be used as potential biocontrol strains for clubroot in cruciferous crops.

### 3.4. Inhibitory Effects of Hz36 and Hk37 on the Development of P. brassicae

**Figure 4:**
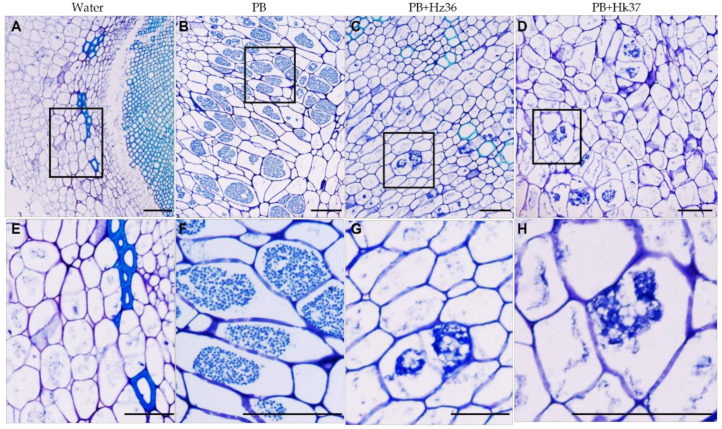
Toluidine blue staining of the root gall cells. (**A**) Rapeseed roots inoculated with water as a negative control. The enlarged picture of the area inside the box is figure (**E**). (**B**) Rapeseed roots inoculated with *P. brassicae* alone. The enlarged picture of the area inside the box is figure (**F**). (**C**) Rapeseed roots co-inoculated with *P. brassicae* and Hz36. The enlarged picture of the area inside the box is figure (**G**). (**D**) Rapeseed roots co-inoculated with *P. brassicae* and Hk37. The enlarged picture of the area inside the box is figure (**H**). Scale bar = 50 μm.

To observe the development of *P. brassicae* in the galls of infected roots, the horizontal sections of rapeseed roots were observed with transmission electron microscopy (TEM) at 25 dpi (Figure S3). The rapeseed root cells inoculated with *P. brassicae* alone were filled with mature resting spores (Figure S3B); while no resting spores were found in the rapeseed root cells treated with PB+Hz36 or PB+Hk37 (Figure S3C,D). At 35 dpi, representative root tissues of toluidine blue-stained paraffin sections were observed with light microscopy. No resting was observed in the negative control root cells (Figure 4A,E), but numerous resting spores were found in positive control root cells (Figure 4B,F). In addition, very few secondary plasmodia were observed on PB+Hz36 and PB+Hk37 treated cell roots (Figure 4C,D,G,H). We determined the content of pathogens in root cells (Figure S4). PB+Hz36 or PB+Hk37 treatment significantly reduced the content of *P. brassicae* in root cells compared with inoculation by PB alone. These observations suggested that Hz36 and Hk37 can inhibit the development of *P. brassicae*.

### 3.5. Inhibitory Effects of Hz36 and Hk37 on Resting Spore Germination

**Figure 5:**
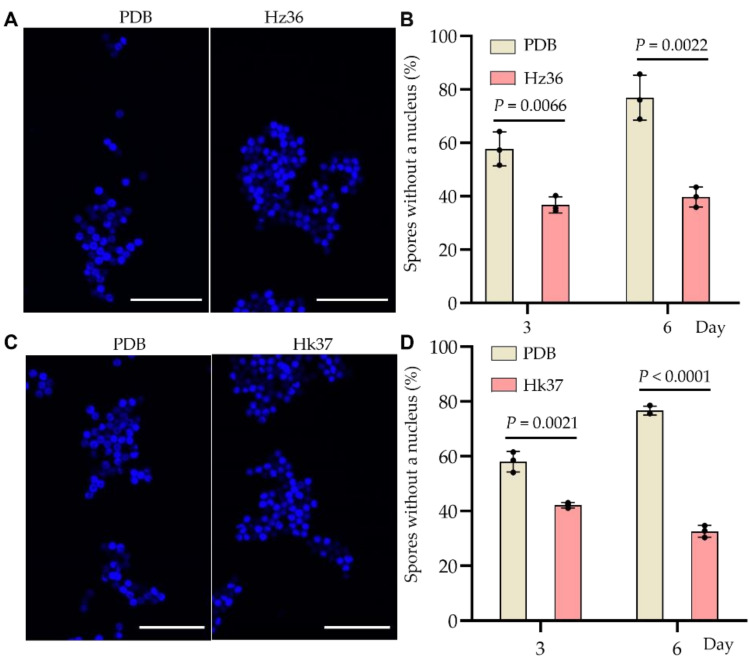
Inhibitory effects of Hz36 and Hk37 fermentation broth on the germination of the *P. brassicae* resting spores. (**A**,**C**) Resting spores of *P. brassicae* treated with the fermentation broth of Hz36 (**A**) and Hk37 (**C**) for 3 days. The pictures show the results of DAPI staining. Fluorescence shows that *P. brassicae* has not germinated, and no fluorescence indicates that the resting spores of *P. brassicae* germinated into primary zoospores, scale bar = 20 μm. (**B**,**D**) Percentage of *P. brassicae* resting spores without a nucleus stained with DAPI after incubation with Hz36 (**B**) and Hk37 (**D**) for 3 days and 6 days. The student’s two-tailed t-test was performed for comparison of means between two data points in B and D, and the data are shown as mean ± s.d. (*n* = 3 biological replicates), black dots show three biological replicates[ST1]. Exact *p*-values for all comparisons are shown in the figure.

To examine the inhibitory effects of Hz36 and Hk37 on resting spore germination of *P. brassicae*, the resting spores were treated with Hz36 or Hk37 fermentation broth for 3 days and 6 days. DAPI staining is a reliable assay system to examine the germination of resting spores [32,33]. The absence or presence of a nucleus in the resting spore could be discriminated under UV-excitation (Figure 5A,C). After 3 days of incubation, the mean percentage of *P. brassicae* resting spores without a nucleus (germination rate) was 36.70% in the Hz36 treatment group, while the percentage was 57.70% in the mock control group (Figure 5B). Similar significant reduction in germination rate was also found after 6 days of incubation (Figure 5A,B). Hk37 showed similar effects to Hz36. The germination rate of resting spores was significantly reduced compared with that of the mock control (Figure 5C,D). The inhibition rate of resting spore germination was 27.20% at 3 days of incubation and 57.40% at 6 days of incubation. Taken together, Hz36 and Hk37 can significantly inhibit the germination of resting spores of *P. brassicae.*

### 3.6. Effects of Hz36 and Hk37 Strains on Rapeseed Seed Germination and Early Root Development

**Figure 6:**
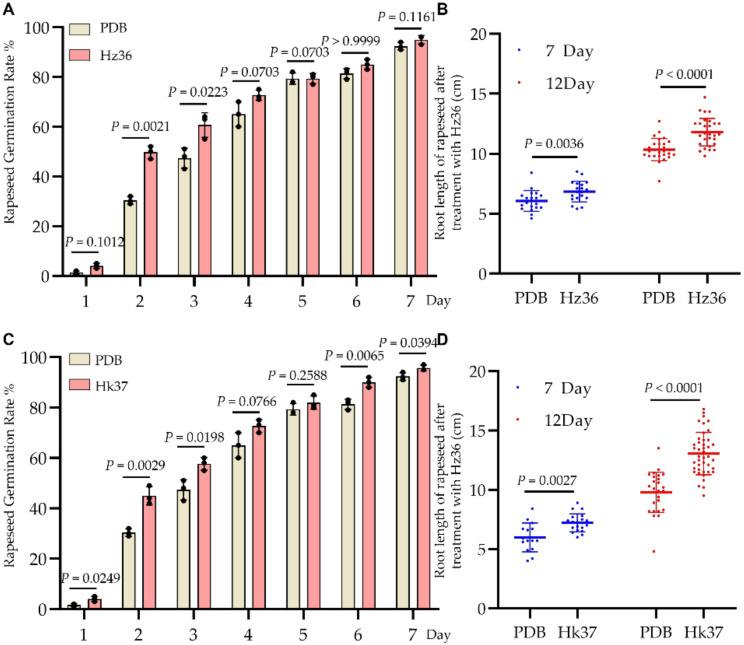
Promotion effect of Hz36 and Hk37 on seeds germination and root length of rapeseed. (**A**) Statistics of the germination rate of rapeseed seeds treated with Hz36 strain for 7 days. (**B**) Root length of rapeseed treated with strain Hz36 at 7 days and 12 days. (**C**) Statistics of the germination rate of rapeseed seeds treated with Hk37 strain for 7 days. (**D**) Root length of rapeseed treated with strain Hk37 at 7 days and 12 days. The student’s two-tailed *t*-test was performed for comparison of means between two data points in A, B, C and D, and the data are shown as mean ± s.d. (*n* = 3 biological replicates in A and C, *n* = 19–31 biological replicates in B and D), black, blue and red dots show biological replicates[ST2]. Exact *p*-values for all comparisons are shown in the figure. Then *p* values in A and C were adjusted using the Benjamini-Hochberg (BH) procedure, BH-adjusted *p* = 0.0126 at the 2 days after Hz36 strain treatment in A, BH-adjusted *p* = 0.0436, *p* = 0.0203, *p* = 0.0436 and *p* = 0.0228 at the 1, 2, 3 and 6 days after Hk37 strain treatment in C, respectively.

Since it has been confirmed that strains Hz36 and Hk37 have biocontrol potential against clubroot, we further evaluated the effect of Hz36 and Hk37 on plant growth. The sterile fermentation filtrate of Hz36 was used to treat rapeseed Huashuang 4 seeds for 6 h, and the seed germination rate was recorded for 1–7 days (Figure 6A). At the first 3 days, the Hz36 treatment group showed significantly higher germination rate than the control group (Figure 6A) but there was no significant difference at 4–7 days (Figure 6A). The root length of rapeseed Huashuang 4 was significantly increased by 11.55% and 12.30% compared with that of the control group at 7 days and 12 days, respectively (Figure 6B). Similar results were obtained for the Hk37 treatment group, as Hk37 treatment enhanced the seed germination rate of rapeseed at 1–3 days and promoted the root growth by 16.20–20.83% (Figure 6C,D). These results demonstrated that Hz36 and Hk37 can enhance the seeds’ germination rate and promote root growth of rapeseed.

## 4. Discussion

Clubroot management has always been a challenge for farmers and pathologists, it is very difficult in control and remove it from the infested fields for the nature of intracellular living parasites and soil-borne characteristics. Chemical as well as classical agronomic measures have not been fully successful to control. Endophytic fungi grow within their host plant tissue without causing visible disease symptoms [36]. They may have beneficial effects on the plant via triggered plant immune responses or promoting plant growth or suppressing plant pathogens [37]. An effective biological control candidate strain requires (1) suppression of the germination the resting spores and/or secondary spores (2) suppression of primary infection of the root hairs and secondary infection of the root cortex; (3) antagonism/competition against the developing pathogen within the host root tissue [4]. In this study, we carried out the isolation and screening of biocontrol endophytic fungi from the inside of roots which infected by *P. brassicae* in severely diseased fields. A total of 44 strains were isolated from the roots, among which 35 belong to *Trichoderma* (Table S1). Hz36 and Hk37 were identified as *T. guizhouense* and *T. koningiopsis*, respectively (Figure 1, Tables S2 and S3, Figures S1 and S2). Strains Hz36 and Hk37 exhibited excellent efficacy to act as biocontrol agents on the model and cruciferous crops (Figure 2 and Figure 3), both of which could significantly inhibit the resting spore germination and development of *P. brassicae* (Figure 4 and Figure 5). activity of various enzymes in plantsIn addition, these two strains could promote seed germination and root growth of rapeseed (Figure 6). These results suggested that Hz36 and Hk37 may serve as new biocontrol agents against clubroot disease. However, the mechanism for the biocontrol effect of strains Hz36 and Hk37 remains unclear, and the biocontrol efficiency needs to be further tested in the field.

Plants always grow in association with numerous microbes. Beneficial microbes can improve the environmental adaptability, defense response, and resource acquisition of plants [38]. With the growing demand for sustainable agriculture and eco-friendly development, the exploration and exploitation of microbial resources are of considerable significance. Biocontrol agents that have been explored are bacteria or fungi. The mechanisms mostly are parasitism, antagonism by toxic/antibiotic secondary metabolites, and/or competition. *Trichoderma* can be used to control many plant diseases, such as rice sheath blight [39], cucumber mosaic [40], and phytophthora blight [41], and confer resistance against more than 20 kinds of pathogenic fungi in 18 genera and a variety of pathogenic bacteria [42]. It can kill other fungi and utilize their nutrients. This behavior is termed mycoparasitism, which is an important mechanism underlying the antagonistic action of *Trichoderma* against pathogens [43,44]. *Trichoderma* can produce various cell wall degrading enzymes, including chitinase, cellulase, xylanase, glucanase and protease, to break down and dissolve pathogens at the contact site of their mycelia, resulting in mycoparasitism [45]. Trichoderma strains and plant results in the modulation of specialized metabolism as well as increased activity of various enzymes in plants. This results in priming against subsequent pathogen attacks in Brassicas and other plants as well [46,47,48,49]. Note the same defensive compounds are required to prevent Trichoderma frombecoming an aggressive colonizer [46]. Apart from the cell wall degrading enzymes that facilitate mycoparasitism, Trichoderma has an arsenal of metabolites that also contribute to their functions as biocontrol agents. For example, the secondary metabolites of *T. harzianum* strain T22 were reported to have antifungal activity against *Leptosphaeria maculans*, *Phytophthora cinnamomi*, and *Botrytis cinerea* [50]. Hz36 and Hk37 might produce some special chemicals, resulting in the inhibition of *P. brassicae* growth and development. However, the detailed mechanism requires further investigation. In past years, a great deal of work has been carried out on the biological control of clubroot. Despite the availability of many microbial control agents against clubroot, little is known about their molecular mechanism. Clarification of the mechanism is required to bring more agents to the approval stage. Recently, the number of studies on the effect of soil microbiome has been rapidly increasing [16]. Microbiome studies of the rhizosphere and endosphere are showing that the microbial communities are complex and important for clubroot development [51,52,53], and microbiome engineering is being vigorously discussed as a biocontrol method [54]. There are many biocontrol agents that show excellent control results that can be achieved in lab trials [13]. Whereas in field trials, those successful control results often cannot be confirmed [55]. It is becoming increasingly clear that the composition of the rhizosphere microbiome is important [56]. Lebreton et al. reported that the microorganism communities of healthy and clubroot-diseased plants have considerable differences [57]. It is still far from an effective clubroot biological control option, but it is becoming possible that microbial communities could make a general contribution to the control of soil-borne plant diseases [58].

## 5. Conclusions

In summary, this study screened and identified two strains of *Trichoderma* with biocontrol potential for clubroot. Strain Hz36 was identified as *T. guizhouense*, and strain Hk37 was identified as *T. koningiopsis*. The fermentation broth of Hz36 could significantly inhibit the germination of resting spores of *P. brassicae*, as well as promote seed germination and root growth of rapeseed. Hz36 and Hk37.could significantly reduce the content of *P. brassicae* and inhibit clubroot development. The results indicate that strains Hz36 and Hk37 have great potential to be used for the biocontrol of clubroot. The biocontrol efficacy of Hz36 and Hk37 in the fields experiment and the biocontrol mechanism needed need to be in the future.

## Data Availability

The data presented in this study are available on request from the corresponding author.

## Supplementary Materials

The following are available online at https://www.mdpi.com/article/10.3390/jof8080777/s1, Figure S1. Identification of Hz36. Maximum likelihood tree based on (*ACT*-*TEF1*-*CAL*-*ITS*) sequences from 40 *Trichoderma*, *T. aggressivum* CBS 100525 and *T. aggressivum* CBS 433.95 were selected as an outgroup. Ex-type strains were highlighted in star. The scale bar = 0.03; Figure S2. Identification of Hk37. Maximum likelihood tree based on (*TEF1*-*CAL*-*ACT*) sequences from 25 *Trichoderma*. *T. hamatum* DAOM 167057 was selected as the outgroup. Ex-type strains were emphasized in star. The scale bar = 0.04; Figure S3. Transmission electron microscopy observation of *P. brassicae* treated with strain Hz36 and Hk37. (A) Rapeseed inoculated with water as a negative control. (B) Rapeseed inoculated with *P. brassicae* alone for 30 days. (C) Rapeseed co-inoculated with *P. brassicae* and Hz36 strain for 30 days. (D) Rapeseed co-inoculated with *P. brassicae* and Hk37 strain for 30 days. The scale bar = 2 μm, PC = plant cell; PCW = plant cell wall; S P = secondary plasmodium; Figure S4. Proportion of the *P. brassicae* in root cells of inoculated with *P. brassicae* alone or co-inoculated, inoculated with water only as a negative control. Results show the means ± s.d. (*n* = 3 biological replicates), black dots show biological replicates, data analyzed with one-way ANOVA by Tukey’s multiple comparisons were conducted using Prism 8. significance set at *p* ≤ 0.05. Different letters (a and b) shown significantly different. Table S1. The information of 44 endophyte fungi isolated from the symptomatic and asymptomatic roots of rapeseed infected by *P. brassicae* in severely infected fields; Table S2. Strains used in Hz36 phylogenetic analysis, and their GenBank numbers; Table S3. Strains used in Hk37 phylogenetic analysis, and their GenBank numbers. Table S4. Primer sequences used in this paper.
