# Supplementary material for: Application of Trichoderma Hz36 and Hk37 as Biocontrol Agents against Clubroot Caused by Plasmodiophora brassicae
Source: J Fungi (Basel). 2022 Jul 26;8(8):777. doi: 10.3390/jof8080777 (PMC9331738; doi:10.3390/jof8080777)
Supplement: Supplementary file 1 [file jof-08-00777-s001.zip › jof-1804414-supplementary.pdf]

Journal of
Fungi

MDPI

Article

Application of *Trichoderma* Hz36 and Hk37 as Biocontrol Agents against Clubroot Caused by *Plasmodiophora brassicae*

Yanli Zhao, Xingfu Chen, Jiasen Cheng, Jiatao Xie, Yang Lin, Daohong Jiang and Yanping Fu * and Tao Chen *

Supplement information:

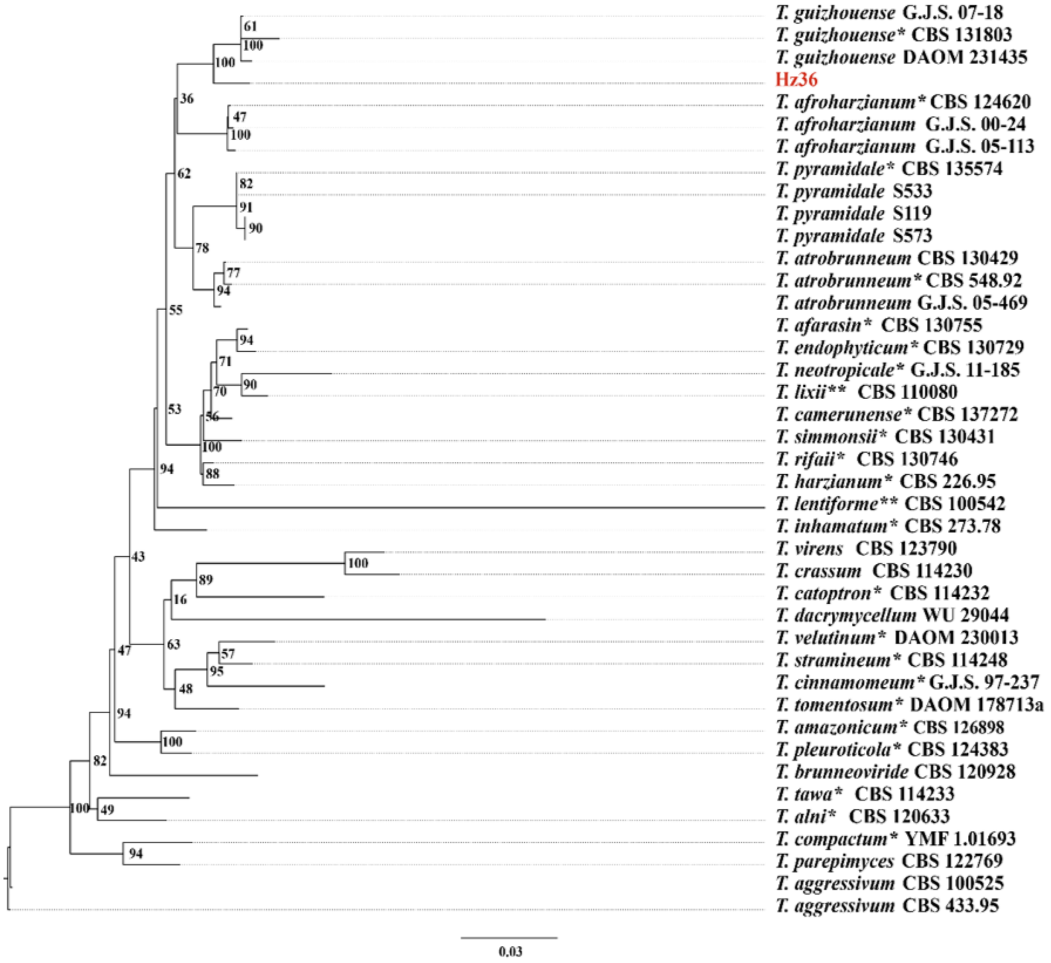

Figure S1. Identification of Hz36. Maximum likelihood tree based on (*ACT-TEF1-CAL-ITS*) sequences from 40 *Trichoderma*, *T. aggressivum* CBS 100525 and *T. aggressivum* CBS 433.95 were selected as an outgroup. Ex-type strains were emphasized in star. Scale bar=0.03.

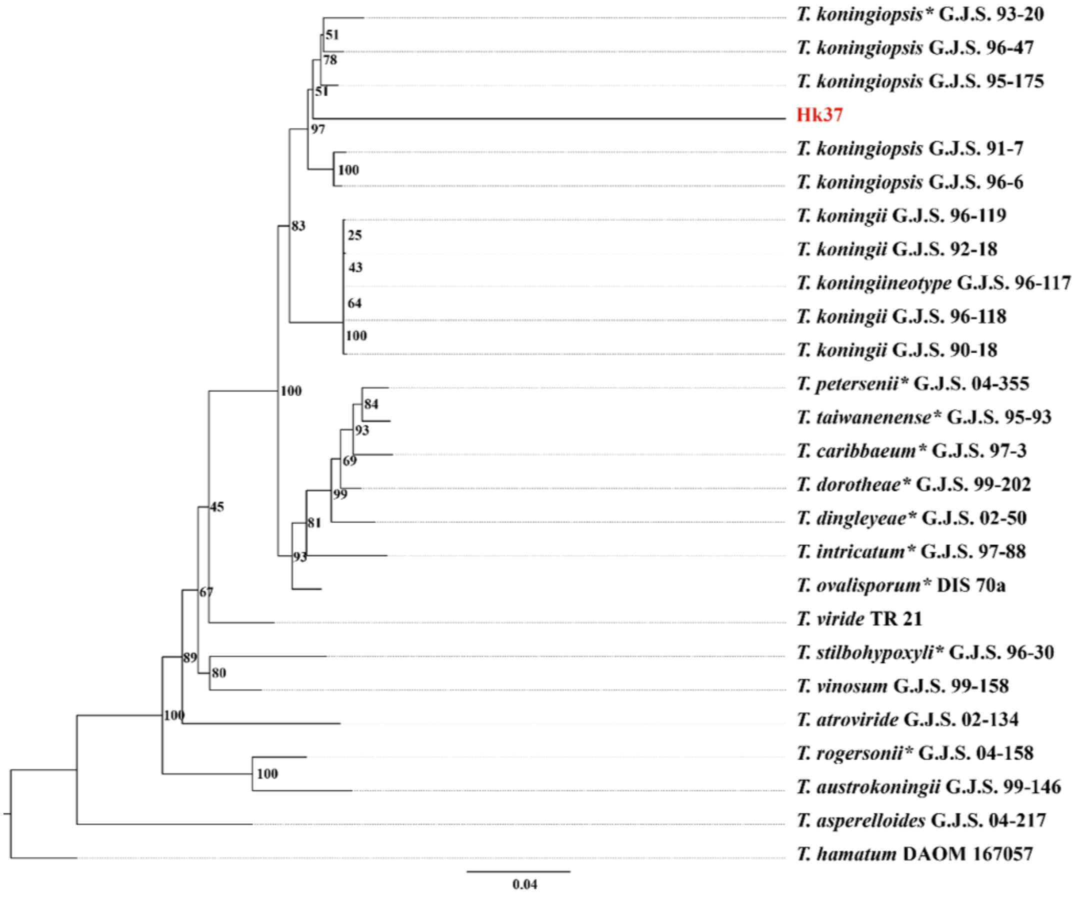

Figure S2. Identification of Hk37. Maximum likelihood tree based on (*TEF1-CAL-ACT*) sequences from 25 *Trichoderma*. *T. hamatum* DAOM 167057 was selected as the outgroup. Ex-type strains were emphasized in star. Scale bar = 0.04.

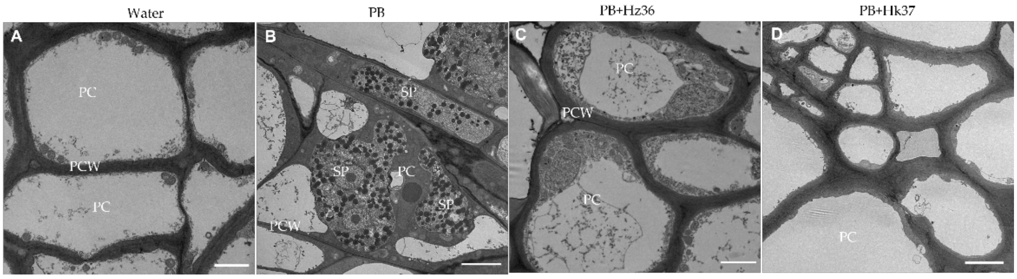

Figure S3. Transmission electron microscopy observation of P. brassicae treated with strain Hz36 and Hk37. (A) Rapeseed inoculated with water as a negative control. (B) Rapeseed inoculated with P. brassicae alone for 30 days. (C) Rapeseed co-inoculated with P. brassicae and Hz36 strain for 30 days. (D) Rapeseed co-inoculated with P. brassicae and Hk37 strain for 30 days. The scale bar = 2 μm, PC = plant cell; PCW = plant cell wall; S P= secondary plasmodium.

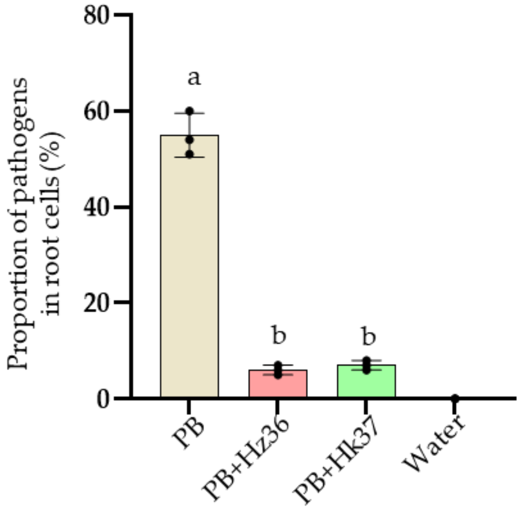

Figure S4. Proportion of *P. brassicae* in root cells of inoculated with *P. brassicae* alone or co-inoculated, inoculated with water only as a negative control. Results show the means ± s.d. (n = 3 biological replicates), data analyzed with one-way ANOVA by Tukey's multiple comparisons were conducted using Prism 8. significance set at $P \leq 0.05$. Different letters (a and b) shown significantly different.

Table S1. The information of 44 endophyte fungi isolated from the symptomatic and asymptomatic roots of rapeseed infected by *P. brassicae* in severely infected fields.

Strain	domain	Phylum	Class	Order	Family	Genus	Species	Biocontrol efficiency
22	Fungi	Ascomycota	Dothideomycetes	*Pleosporales*	*Pleosporaceae*	*Alternaria*	*Alternaria tenuissima*	NA
44	Fungi	Ascomycota	Sordariomycetes	*Hypocreales*	*Nectriaceae*	*Fusarium*	*Fusarium proliferatum*	NA
7	Fungi	Ascomycota	Sordariomycetes	*Hypocreales*	*Hypocreaceae*	*Hypocrea*	*Hypocrea lixii*	32.13%
38	Fungi	Ascomycota	Sordariomycetes	*Hypocreales*	*Hypocreaceae*	*Hypocrea*	*Hypocrea lixii*	10.92%
15	Fungi	Ascomycota	Sordariomycetes	*Hypocreales*	*Hypocreaceae*	*Hypocrea*	unclassified Hypocrea	NA
3	Fungi	Mucoromyceta	Mucoromycetes	*Mucorales*	*Mucoraceae*	*Mucor*	*Mucor circinelloides*	NA
2	Fungi	Mucoromyceta	Mucoromycetes	*Mucorales*	*Mucoraceae*	*Mucor*	*Mucor hiemalis*	NA
9	Fungi	Mucoromyceta	Mucoromycetes	*Mucorales*	*Mucoraceae*	*Mucor*	*Mucor hiemalis*	NA
10	Fungi	Mucoromyceta	Mucoromycetes	*Mucorales*	*Mucoraceae*	*Mucor*	*Mucor hiemalis*	NA
16	Fungi	Mucoromyceta	Mucoromycetes	*Mucorales*	*Mucoraceae*	*Mucor*	*M. racemosus*	NA
39	Fungi	Oomycota	Oomycetes	*Pythiales*	*Pythiaceae*	*Pythium*	*Pythium coloratum*	NA
40	Fungi	Oomycota	Oomycetes	*Pythiales*	*Pythiaceae*	*Pythium*	*Pythium coloratum*	NA
41	Fungi	Oomycota	Oomycetes	*Pythiales*	*Pythiaceae*	*Pythium*	*Pythiumcoloratum*	NA
32	Fungi	Ascomycota	Sordariomycetes	*Hypocreales*	*Hypocreaceae*	*Trichoderma*	*Trichoderma atroviride*	NA
34	Fungi	Ascomycota	Sordariomycetes	*Hypocreales*	*Hypocreaceae*	*Trichoderma*	*Trichoderma atroviride*	NA
17	Fungi	Ascomycota	Sordariomycetes	*Hypocreales*	*Hypocreaceae*	*Trichoderma*	*Trichoderma atroviridis*	NA
19	Fungi	Ascomycota	Sordariomycetes	*Hypocreales*	*Hypocreaceae*	*Trichoderma*	*Trichoderma atroviridis*	NA
20	Fungi	Ascomycota	Sordariomycetes	*Hypocreales*	*Hypocreaceae*	*Trichoderma*	*Trichoderma atroviridis*	NA
23	Fungi	Ascomycota	Sordariomycetes	*Hypocreales*	*Hypocreaceae*	*Trichoderma*	*Trichoderma atroviridis*	NA
24	Fungi	Ascomycota	Sordariomycetes	*Hypocreales*	*Hypocreaceae*	*Trichoderma*	*Trichoderma atroviridis*	NA
33	Fungi	Ascomycota	Sordariomycetes	*Hypocreales*	*Hypocreaceae*	*Trichoderma*	*Trichoderma atroviridis*	NA
5	Fungi	Ascomycota	Sordariomycetes	*Hypocreales*	*Hypocreaceae*	*Trichoderma*	*Trichoderma atroviridis*	NA
6	Fungi	Ascomycota	Sordariomycetes	*Hypocreales*	*Hypocreaceae*	*Trichoderma*	*Trichoderma atroviridis*	NA
11	Fungi	Ascomycota	Sordariomycetes	*Hypocreales*	*Hypocreaceae*	*Trichoderma*	*Trichoderma atroviridis*	NA
12	Fungi	Ascomycota	Sordariomycetes	*Hypocreales*	*Hypocreaceae*	*Trichoderma*	*Trichoderma atroviridis*	NA
13	Fungi	Ascomycota	Sordariomycetes	*Hypocreales*	*Hypocreaceae*	*Trichoderma*	*Trichoderma atroviridis*	NA
14	Fungi	Ascomycota	Sordariomycetes	*Hypocreales*	*Hypocreaceae*	*Trichoderma*	*Trichoderma atroviridis*	19.39%
25	Fungi	Ascomycota	Sordariomycetes	*Hypocreales*	*Hypocreaceae*	*Trichoderma*	*Trichoderma atroviridis*	NA
26	Fungi	Ascomycota	Sordariomycetes	*Hypocreales*	*Hypocreaceae*	*Trichoderma*	*Trichoderma atroviridis*	NA
27	Fungi	Ascomycota	Sordariomycetes	*Hypocreales*	*Hypocreaceae*	*Trichoderma*	*Trichoderma atroviridis*	-10.28%
28	Fungi	Ascomycota	Sordariomycetes	*Hypocreales*	*Hypocreaceae*	*Trichoderma*	*Trichoderma atroviridis*	NA
29	Fungi	Ascomycota	Sordariomycetes	*Hypocreales*	*Hypocreaceae*	*Trichoderma*	*Trichoderma atroviridis*	NA
43	Fungi	Ascomycota	Sordariomycetes	*Hypocreales*	*Hypocreaceae*	*Trichoderma*	*Trichoderma atroviridis*	NA
36	Fungi	Ascomycota	Sordariomycetes	*Hypocreales*	*Hypocreaceae*	*Trichoderma*	*Trichoderma guizhouense*	52.18%
1	Fungi	Ascomycota	Sordariomycetes	*Hypocreales*	*Hypocreaceae*	*Trichoderma*	*Trichoderma harzianum*	NA
8	Fungi	Ascomycota	Sordariomycetes	*Hypocreales*	*Hypocreaceae*	*Trichoderma*	*Trichoderma harzianum*	NA
21	Fungi	Ascomycota	Sordariomycetes	*Hypocreales*	*Hypocreaceae*	*Trichoderma*	*Trichoderma harzianum*	NA
42	Fungi	Ascomycota	Sordariomycetes	*Hypocreales*	*Hypocreaceae*	*Trichoderma*	*Trichoderma harzianum*	10%
37	Fungi	Ascomycota	Sordariomycetes	*Hypocreales*	*Hypocreaceae*	*Trichoderma*	*Trichoderma koningiopsis*	68.01%
4	Fungi	Ascomycota	Sordariomycetes	*Hypocreales*	*Hypocreaceae*	*Trichoderma*	*Trichoderma viride*	NA
18	Fungi	Ascomycota	Sordariomycetes	*Hypocreales*	*Hypocreaceae*	*Trichoderma*	*Trichoderma viride*	5%
30	Fungi	Ascomycota	Sordariomycetes	*Hypocreales*	*Hypocreaceae*	*Trichoderma*	*Trichoderma viride*	NA
31	Fungi	Ascomycota	Sordariomycetes	*Hypocreales*	*Hypocreaceae*	*Trichoderma*	*Trichoderma viride*	NA
35	Fungi	Ascomycota	Sordariomycetes	*Hypocreales*	*Hypocreaceae*	*Trichoderma*	*Trichoderma viride*	NA

Note: Fonts marked in red indicated the strain was selected against P. brassicae in Arabidposis in the green house condition, inoculated with P. brassicae for 21 days.

Table S2. Strains used in Hz36 phylogenetic analysis and their GenBank numbers.

Species	Culture number	GenBank accession number			
		ACT	*TEF1*	*CAL*	ITS
*Trichoderma afarasin**	CBS 130755	FJ442536	AF348093	FJ442388	AY027784
*Trichoderma afroharzianum**	CBS 124620	-	FJ463301	FJ442370	FJ442265
Trichoderma afroharzianum	G.J.S. 00-24	FJ442488	AF443940	AF442880	AF443922
Trichoderma afroharzianum	G.J.S. 05-113	FJ442571	FJ463378	FJ442371	FJ442235
Trichoderma aggressivum	CBS 100525	FJ442433	AF348095	AF442859	AF057600
Trichoderma aggressivum	CBS 433.95	FJ442436	AF348097	FJ442279	FJ442605
*Trichoderma alni**	CBS 120633	GQ250315	EU498312	EU498326	EU518651
*Trichoderma amazonicum**	CBS 126898	-	HM142376	-	HM142358
*Trichoderma atrobrunneum**	CBS 548.92	FJ442528	AF443942	AF442883	AF443924
Trichoderma atrobrunneum	G.J.S. 05-469	FJ442502	FJ463304	FJ442340	FJ442629
Trichoderma atrobrunneum	CBS 130429	FJ442525	AF443943	AF442886	AF443926
Trichoderma brunneoviride	CBS 120928	-	EU498318	EU498330	EU518661
*Trichoderma camerunense**	CBS 137272	FJ442537	AF348107	AF442875	AY027780
*Trichoderma catoptron**	CBS 114232	FJ442584	AY737726	FJ442387	AY737766
Trichoderma cinnamomeum	G.J.S. 97-237	FJ442582	AY737732	JN133524	AY737759
*Trichoderma compactum**	YMF 1.01693	-	AY941824	-	-
Trichoderma crassum	CBS 114230	-	JN133572	-	-
Trichoderma dacrymycellum	WU 29044	-	FJ860633	-	FJ860749
*Trichoderma endophyticum**	CBS 130729	FJ442445	FJ463319	FJ442292	FJ442243
*Trichoderma guizhouense**	CBS 131803	-	JN215484	-	JN191311
Trichoderma guizhouense	DAOM 231435	-	EF191321	FJ577721	EF191296
Trichoderma guizhouense	G.J.S. 07-18	FJ442521	FJ463390	FJ442355	FJ442641
*Trichoderma harzianum**	CBS 226.95	FJ442567	AF348101	AF442864	AJ222720
*Trichoderma inhamatum**	CBS 273.78	FJ442561	AF348099	AF442891	FJ442680
*Trichoderma lentiforme***	CBS 100542	AF469193	AF469195	AF469191	AF469189
*Trichoderma lixii***	CBS 110080	FJ442533	AF443938	AF442872	AF443920
*Trichoderma neotropicale**	G.J.S. 11-185	KP115268	HQ022771	KP115279	HQ022407
Trichoderma parepimyces	CBS 122769	-	FJ860664	-	FJ860800
*Trichoderma pleuroticola**	CBS 124383	JN133598	HM142381	JN133538	HM142362
*Trichoderma pyramidale**	CBS 135574	-	KJ665699	-	-
Trichoderma pyramidale	S119	-	KJ665696	-	-
Trichoderma pyramidale	S533	-	KJ665697	-	-
Trichoderma pyramidale	S573	-	KJ665698	-	-
*Trichoderma rifaii**	CBS 130746	FJ442474	FJ463324	-	FJ442663
*Trichoderma simmonsii**	CBS 130431	FJ442526	AF443935	AF442869	AF443917
*Trichoderma stramineum**	CBS 114248	FJ442583	AY737746	FJ442386	AY737765
*Trichoderma tawa**	CBS 114233	FJ442570	FJ463313	FJ442406	AY737756
*Trichoderma tomentosum**	DAOM 178713a	JN133612	AY750882	JN133548	EU330958
*Trichoderma velutinum**	DAOM 230013	JN133614	AY937415	JN133550	AF149873
Trichoderma virens	CBS 123790	FJ442589	AY750894	FJ442404	DQ083023

Note: Ex-type strains were emphasized in star.

Table S3. Strains used in Hk37 phylogenetic analysis and their GenBank numbers.

Species	Strain	GenBank accession number		
		TEF1	*CAL*	*ACT*
Trichoderma atroviride	G.J.S. 02-134	DQ307547	-	-
*Trichoderma austrokoningii**	G.J.S. 99-146	DQ307561	DQ379001	DQ367716
Trichoderma asperellum	G.J.S. 04-217	DQ381958	DQ379002	DQ333564
Trichoderma caribbaeum *	G.J.S. 97-3	DQ284977	DQ367688	DQ328607
*Trichoderma dingleyae**	G.J.S. 02-50	DQ284978	DQ370461	DQ367718
*Trichoderma dorotheae**	G.J.S. 99-202	DQ307536	DQ367706	DQ323454
Trichoderma hamatum	DAOM 167057	AF456911	DQ122154	DQ111962
Trichoderma intricatum *	G.J.S. 97-88	AY376060	DQ367680	AY376684
Trichoderma koningii	G.J.S. 96-117	AF456909	DQ367686	DQ341180
Trichoderma koningii	G.J.S. 90-18	DQ289007	DQ370463	DQ341181
Trichoderma koningii	G.J.S. 92-18	DQ289005	DQ370458	DQ367712
Trichoderma koningii	G.J.S. 96-118	DQ288996	DQ367683	DQ341178
Trichoderma koningii	G.J.S. 96-119	DQ289003	DQ367681	DQ341179
Trichoderma koningiopsis	G.J.S. 91-6	DQ307539	DQ367705	DQ323446
Trichoderma koningiopsis	G.J.S. 91-7	DQ284969	DQ367689	DQ323449
Trichoderma koningiopsis	G.J.S. 95-175	AF456910	DQ367707	DQ323448
Trichoderma koningiopsis	G.J.S. 96-47	AY376044	DQ367670	AY376677
*Trichoderma koningiopsis**	G.J.S. 93-20	DQ284966	DQ367694	DQ381954
*Trichoderma ovalisporum**	DIS 70a	AY376037	DQ379005	AY376671
*Trichoderma petersenii**	G.J.S. 04-355	DQ284980	DQ367691	DQ333570
*Trichoderma rogersonii**	G.J.S. 04-158	DQ307563	DQ370460	DQ333567
*Trichoderma stilbohypoxyli**	G.J.S. 96-30	DQ109546	DQ122161	DQ111967
*Trichoderma taiwanense**	G.J.S. 95-93	DQ284973	DQ367685	DQ323455
Trichoderma viride	TR 21	AY376054	DQ122165	AY376681
Hypocrea vinosa	G.J.S. 99-158	AY376047	DQ122158	AY376680

Note: Ex-type strains were emphasized in star.

Table S4. Primer sequences used in this paper.

**Primer name**	**Primers sequence (5’→3’)**	**Target**
ITS1	TCCGTAGGTGAACCTGCGG	ITS
ITS4	TCCTCCGCTTATTGATATGC	ITS
ITS5	GGAAGTAAAAGTCGTAACAAGG	ITS
Tact1	TGGCACCACACCTTCTACAATGA	*ACT*
Tact2	TCTCCTTCTGCATACGGTCGGA	*ACT*
EF1-728F	CATCGAGAAGTTCGAGAAGG	*TEF1*
EF1-1R	GCCATCCTTGGGAGATACCAGC	*TEF2*
CAL-228F	GAGTTCAAGGAGGCCTTCTCCC	*CAL*
CAL-737R	CATCTTTCTGGCCATCATGG	*CAL*
Pbactin-F	CACCGACTACCTGATGAA	qRT-PCR
Pbactin-R	CAGCTTCTCCTTGATGTC	qRT-PCR
Atactin-F	AATCCACGAGACAACCTA	qRT-PCR
Atactin-R	AGCGATACCTGAGAACATA	qRT-PCR
Bnactin-F	TGAAGATCAAGGTGGTCGCA	qRT-PCR
Bnactin-R	GAAGGCAGAAACACTTAGAAG	qRT-PCR
